# Objective and subjective sleep disorders in automated peritoneal dialysis

**DOI:** 10.1186/s40697-016-0093-x

**Published:** 2016-02-17

**Authors:** Maria-Eleni Roumelioti, Christos Argyropoulos, Vernon Shane Pankratz, Manisha Jhamb, Filitsa H. Bender, Daniel J. Buysse, Patrick Strollo, Mark L. Unruh

**Affiliations:** Nephrology Division, Department of Medicine, University of New Mexico, 901 University Blvd. SE, Suite 150, MSC 04-2785, Albuquerque, NM 87106 USA; Renal-Electrolyte Division, Department of Medicine, University of Pittsburgh Medical Center, Pittsburgh, PA USA; Department of Psychiatry, University of Pittsburgh, Pittsburgh, PA USA; Division of Pulmonary, Allergy and Critical Care Medicine, Department of Medicine, University of Pittsburgh, Pittsburgh, PA USA

**Keywords:** Sleep-disordered breathing, Hypoxemia, Sleep quality, Periodic limb movements, Depression, Automated peritoneal dialysis, Chronic kidney disease, Hemodialysis

## Abstract

**Background:**

Automated peritoneal dialysis (APD) is one of the fastest growing dialysis modalities. It is unknown whether sleep and mood are disturbed while performing repeated overnight exchanges.

**Objectives:**

In this report, we aim to describe and compare the prevalence of sleep-disordered breathing (SDB), periodic limb movements (PLMS), poor sleep quality (SQ), and depression among APD patients compared with stages 3b–5 (estimated glomerular filtration rate ≤44 ml/min/1.73 m2) chronic kidney disease (CKD) and hemodialysis (HD) patients.

**Design:**

This is a cross-sectional, descriptive study.

**Setting:**

Study participants were recruited from outpatient nephrology clinics, local dialysis centers, and the Thomas E. Starzl Transplant Institute in Western Pennsylvania between April 2004 and July 2009.

**Patients:**

There were 186 participants in this study including 22 APD patients, 89 CKD patients, and 75 HD patients.

**Measurements:**

In-home polysomnography was performed and two questionnaires were completed, the Pittsburgh Sleep Quality Index (PSQI) and the Patient Health Questionnaire-9 (PHQ-9).

**Methods:**

SDB and PLMS were quantified by in-home unattended polysomnography; poor SQ was defined by a score >5 on the PSQI, and the presence of moderate to severe depression was defined by a score >5 on the PHQ-9.

**Results:**

The APD patients had a median age of 37.5 years, were predominantly female (72.7 %), and had a median body mass index (BMI) of 23.8 kg/m2. In univariate analyses, APD patients had significantly lower apnea-hypopnea index compared to HD patients by 12.2 points (likelihood ratio test p = 0.008) and revealed the least percent of TST with nocturnal hypoxemia compared to CKD patients by 2.7 points, respectively (likelihood ratio test p = 0.01). The APD group had also significantly greater stages 3 to 4 sleep compared to the CKD patients by 8.6 points (likelihood ratio test p = 0.009). In multivariate analyses and after adjustment for age, gender, race, and BMI, both APD and HD patients had higher average PSQI scores than CKD patients by 2.54 and 2.22 points, respectively (likelihood ratio test p = 0.005). No other comparisons of sleep parameters among groups reached statistical significance.

**Limitations:**

The limitations of this study are the small sample size of the APD population and the demographic and clinical differences among the three study groups.

**Conclusions:**

Despite differences in univariate analyses, after multivariate adjustment, APD patients had similar sleep parameters and sleep architecture and as poor SQ and symptoms of depression as HD patients. Future studies with larger APD cohorts are needed.

## ABRÉGÉ

**Mise en contexte:**

La dialyse péritonéale automatisée (DPA) est l’une des méthodes de traitement pour l’insuffisance rénale terminale connaissant la plus forte croissance. Toutefois, l’influence de la pratique répétée de cette procédure sur la qualité du sommeil et sur l’humeur du patient demeure peu documentée.

**Objectifs de l’étude:**

L’étude visait à caractériser et à définir la prévalence de troubles respiratoires du sommeil, de mouvements involontaires des membres, d’effets sur la qualité du sommeil et de symptômes dépressifs chez les patients sous DPA en comparaison avec la prévalence de ces mêmes paramètres chez les patients souffrant d’insuffisance rénale chronique (eDFG à ≤44 ml/min/1.73 m^2^) et chez les patients sous hémodialyse.

**Cadre et type d’étude:**

Il s’agit d’une étude transversale descriptive qui s’est tenue dans l’ouest de la Pennsylvanie (États-Unis) entre avril 2004 et juillet 2009. Le recrutement des participants s’est effectué au sein des patients en consultation externe des cliniques de néphrologie, dans les centres de dialyses régionaux et au *Thomas E. Starzl Transplant Institute*.

**Participants:**

Un total de 186 patients a pris part à cette étude, parmi lesquels on comptait 22 patients sous traitement par DPA, 89 patients en situation d’insuffisance rénale chronique et 75 patients sous hémodialyse.

**Mesures:**

La cueillette des données incluait des mesures de polysomnographie effectuées à domicile par les patients ainsi que les réponses fournies à deux questionnaires : le *Pittsburgh Sleep Quality Index* (PSQI) et le *Patient Health Questionnaire-*9 (PHQ-9).

**Résultats:**

La cohorte de patients sous DPA se constituait en majorité de femmes (72,3 %) de 37,5 ans d’âge médian et dont l’indice de masse corporelle (IMC) se situait en moyenne à 23,8 kg/m^2^. Les analyses univariées ont démontré un index apnées-hypopnées inférieur de 12,2 points (p = 0,0008) et un plus faible pourcentage de troubles du sommeil avec hypoxémie nocturne par 2,7 points (p = 0,01) chez les patients sous DPA en comparaison avec les patients sous hémodialyse. Les analyses multivariées, après correction en fonction de l’âge, du sexe, de l’origine ethnique et de l’IMC des participants, ont quant à elles démontré que les patients sous DPA et sous hémodialyse ont obtenu des résultats plus élevés au questionnaire PSQI que les patients souffrant d’insuffisance rénale chronique, avec des moyennes supérieures de 2,54 et 2,22 points respectivement (p = 0,005). La comparaison entre les différents groupes en ce qui concerne les autres paramètres mesurés n’a pas atteint le degré de signification statistique.

**Limites de l’étude:**

Les données recueillies au cours de cette étude concernent un faible échantillon de patients sous DPA. De plus, en raison des grandes différences notées dans les données cliniques et démographiques des trois groupes de patients qui la constituaient, la cohorte étudiée manquait d’uniformité.

**Conclusions:**

En dépit des différences notées dans les analyses univariées et de l’obligation d’effectuer des corrections dans les analyses multivariées, les résultats montrent que les patients sous DPA ne présentent pas de différences significatives pour les paramètres de sommeil mesurés. Les patients sous DPA maintiennent des paramètres et une architecture de sommeil semblables à ceux mesurés chez les patients sous traitement par hémodialyse : la qualité du sommeil demeure faible, mais ils ne présentent pas plus de symptômes de dépression. Des études de plus grande envergure sont requises afin de mieux définir l’influence de la DPA sur la qualité du sommeil et sur l’humeur des patients qui la pratiquent.

Sleep-disordered breathing and periodic limb movements are two of the most frequent sleep disorders among patients undergoing peritoneal dialysis, while depression is a major factor contributing to their poor sleep quality. Sleep and mood assessment among APD patients has been overlooked despite the substantial health burden associated with sleep disturbances and depression. This report adds to the pre-existing knowledge by examining by objective and subjective means sleep and mood among automated peritoneal dialysis patients and by comparing them to stages 3b–5 chronic kidney disease and hemodialysis patients.

## Background

Symptoms of poor sleep are commonly reported among patients with chronic kidney disease (CKD) and end-stage renal disease (ESRD) dependent on dialysis. However, most reports focus on patients undergoing hemodialysis (HD). This overlooks patients performing automated peritoneal dialysis (APD), which is the fastest growing dialysis modality, and unique in that dialysis is performed at home largely during sleep. In patients undergoing peritoneal dialysis (PD), sleep-disordered breathing (SDB), restless legs syndrome, and periodic limb movements (PLMS) in sleep are thought to be the most common sleep disorders [1]. Negative emotional states (e.g., depression), somatic symptoms, and treatment-related issues (e.g., cycler alarms) are some of the factors contributing to poor sleep quality (SQ) in this vulnerable patient population [2, 3].

While treatment-related aspects of APD may negatively influence SQ, SDB is the most frequently reported sleep disorder in ESRD (50–80 % in most studies) [2, 4–7] and has been related mostly to chronic fluid retention [8], uremia [8], and abdominal dialysate bulk load during the night exchanges [6]. APD patients seem to have less severe SDB compared to continuous ambulatory PD patients, perhaps due to more effective ultrafiltration and solute clearance [4]. PLMS are also highly prevalent (40–70 %) in patients on maintenance dialysis (PD or HD) [9–12] and have been associated with SDB and mortality in these patients [13, 14]. Although depression is common among patients on chronic dialysis [15, 16] and may be a significant contributor to their poor SQ, previous studies comparing dialysis modalities as well as examining the emotional well-being and the presence of depression among PD patients have reported contradictory findings.

Sleep assessment among APD patients has been overlooked despite the substantial health burden associated with sleep disturbances. Sleep disorders can lead to excessive daytime sleepiness [17] and unintentional napping during the day [18], reduced sleep quantity and quality [2], psychological distress [17], cognitive dysfunction [19], reduced quality of life [20], and chronic inflammation [21, 22] and have been associated with increased cardiovascular and all-cause mortality [23] in PD patients. These disorders have also been linked to greater use of health services, increased use of hypnotics, and reduced functional capabilities [24]. Poor self-reported SQ occurs in 47–80 % of PD patients [25–27] and correlates significantly with psychosocial problems, marital status, educational background, and patients’ perceptions of quality of life [28]. Few prior studies have examined sleep disorders in patients on PD and compared to HD and advanced CKD patients [2, 29]. These studies have largely focused on symptomatic continuous ambulatory PD patients and overlooked important outcomes such as sleep efficiency and sleep arousals.

In this report, we aimed to characterize SDB, PLMS, SQ, and depression among cycler-assisted APD patients. We also compared those sleep parameters against stages 3b–5 CKD and to HD patients.

## Methods

### Study setting, samples, and design

#### Patients

For this report, 22 APD, 89 non-dialysis-dependent CKD patients, and 75 HD patients were enrolled from outpatient nephrology clinics, local dialysis centers, and the Thomas E. Starzl Transplant Institute in Western Pennsylvania between April 2004 and July 2009. Patients were eligible to participate if they were >18 years and had advanced CKD (Modification of Diet in Renal Disease-derived estimated glomerular filtration rate, estimated glomerular filtration rate (eGFR) ≤44 ml/min/1.73 m^2^). Patients were excluded for use of continuous positive airway pressure and for active medical or psychiatric disease (e.g., unstable angina, alcohol abuse). Potential study participants were approached and informed about the sleep study by clinical staff who was directly involved in their care or by study coordinators themselves while visiting their clinic. No “cold-calling” occurred for study recruitment. If interested in research study participation, the clinician would instruct the participant to either contact the research team directly for additional information or the participant would directly provide a signed IRB-approved written consent form and a signed written HIPPA authorization form to our research staff.

The 89 CKD and 75 HD remaining patients who participated in the sleep studies are included in this report. Of note, much of the data from these two comparison groups have been published in a comparison with controls from the Sleep Strategies Concentrating on Risk Evaluation (SCORE) study [30]. Patient preference determined the night of polysomnography (PSG) conduct relative to their HD day. Of the 67 HD patients with available data, 31 (41.3 %) and 36 (48 %) patients were studied the evening after and before their session, respectively. Of the 57 HD patients with available shift data, 40 were in the morning (5:30 to 10:00 a.m.), 16 in the afternoon (10:00 a.m. to 3:30 p.m.), and 1 in the evening shift (3:30 to 5:30 p.m.).

The study was approved by the University of Pittsburgh IRB, and all participants provided written informed consent.

### Data collection

Baseline data collection for all participants included a brief standardized health interview, questionnaire administration, assessment of antihypertensives (total number used at the time of the study), systolic and diastolic blood pressure before the PSG assessment, weight, height, neck and waist circumference, and unattended home PSG.

In addition, two measurements of systolic and diastolic blood pressure were performed within at least 2–3 min before each PSG study using an automated cuff. If there was an over 4-mmHg discrepancy between the two cuff readings, a third measurement was performed. Serum creatinine, eGFR, and serum glucose within 6 months from the date of the study were also recorded for all groups.

### Sleep assessment—polysomnography

Unattended in-home PSG was performed using an ambulatory Compumedics Siesta monitor (Charlotte, North Carolina) at habitual sleep times. Patients’ preference determined the night of PSG conduction. APD patients also performed their therapy on the same night. The sleep study montage included bilateral central and occipital electroencephalogram (EEG) channels, bilateral electrooculograms (EOGs), bipolar submentalis electromyograms (EMGs), and one channel of electrocardiogram (ECG) recording. Bipolar ECG and position sensors were used to monitor heart rate and body position, respectively. On the night of PSG, participants were also monitored for respiratory parameters, nasal pressure, and for abdominal and thoracic effort using finger pulse oximetry (Nonin, Minneapolis, MN), nasal-oral thermocouple, and inductance plethysmography, respectively. High-frequency filter settings were 100 Hz for EEG and EOG and 70 Hz for EMG. Low-frequency filter settings were 0.3 Hz for EEG and 10 Hz for EMG [31].

### Scoring of polysomnograms—sleep parameter definitions

Centrally trained PSG technologists scored sleep records for all study groups according to the Rechtschaffen and Kales guidelines using standard sleep stage scoring criteria for each 20-s epoch [32]. All scorers were blinded to the renal function of the patients. Standard definitions were used to identify apneas and hypopneas; oximetry readings were used to quantify average and minimum oxyhemoglobin saturation levels. Apnea was defined as a complete or an almost complete (≤25 % of baseline) airflow cessation, measured by the amplitude of the ≥10-s nasal pressure signal. Hypopnea was defined as a ≥10-s abnormal respiratory event with ≥30 % airflow reduction (compared to baseline) and was associated with ≥4 % oxyhemoglobin desaturation. Limb movements were defined as periodic if they were separated by at least 5 s and not more than 90 s.

PSG outcome variables in the analysis included total sleep time (sleep time excluding periods of wakefulness during the night); sleep efficiency (percentage of total sleep time as a proportion of the total recording duration); parameters of sleep architecture (percentage of total sleep time spent in non-rapid eye movement (NREM) stage 1, stage 2, and stages 3–4 and rapid eye movement (REM) sleep); apnea-hypopnea index (AHI, number of apneas and hypopneas/hour of sleep); micro-arousal index (number of microarousals/hour of sleep); nocturnal hypoxemia (percent of total sleep time with oxyhemoglobin saturation <90 %); [33] and Periodic Limb Movement Index (PLMI, number of PLMS/h of sleep). Moderate to severe SDB was defined as having AHI ≥15. The presence of PLMS was defined as having a PLMI ≥5.

### Sleep quality

Participants also completed the Pittsburgh Sleep Quality Index (PSQI), which is a self-rated questionnaire for evaluating subjective SQ. The PSQI includes 18 questions regarding the subjects’ habitual sleep over the past 1 month that are combined into seven clinically derived component scores, each weighted equally from 0 to 3. The seven component scores are added to obtain a global score ranging from 0 to 21. The PSQI has good internal consistency, test-retest reliability, discriminant validity, and responsiveness to treatments and has been used in the ESRD population [34]. PSQI scores >5 reflect poor SQ.

### Depression

The Patient Health Questionnaire-9 (PHQ-9) is a self-administered, established questionnaire that covers symptoms of major depressive disorder, as well as their subsyndromal variants. The PHQ-9 consists of nine criteria for depression from the Diagnostic and Statistical Manual of Mental Disorders, fourth edition (DSM-IV). PHQ-9 severity is calculated by assigning scores of 0, 1, 2, and 3 to the response categories of: not at all, several days, more than half the days, and nearly every day, respectively, for each of the nine items. PHQ-9 total score for the nine items ranges from 0 to 27. Scores of 5, 10, 15, and 20 represent cut points for mild, moderate, moderately severe, and severe depression, respectively. The PHQ-9 is comparable or superior in operating characteristics and valid as both a diagnostic and severity measure [35]. Sensitivity to change has also been confirmed [36]. The PHQ-9 was completed by all consenting individuals, and any findings of a positive score for depression and/or suicidal ideation were reported promptly to the participant’s primary physician.

### Statistical analysis

The study population characteristics and the various sleep parameters are presented as medians and interquartile ranges (25th and 75th percentiles) for continuous variables or as frequencies and percentages for categorical variables. Non-parametric Kruskal-Wallis tests were used to examine the initial statistical significance of the differences among the study groups. For patient characteristics and sleep parameters that were significantly different by the global Kruskal-Wallis test, Mann-Whitney *U* tests were performed for pairwise comparisons of study groups. In order to examine among-group differences after accounting for demographic and clinical differences among the study groups, we performed analyses that compared the study groups while adjusting for differences in patient characteristics using analysis of covariance approaches. For each of the variables of interest, we identified a transformation, typically square root or logarithmic, that provided a data scale that better met modeling assumptions. We performed analysis of covariance for each of these transformed variables and tested for differences among study groups after adjusting for age, sex, race, and body mass index (BMI) in our primary adjusted analyses. We obtained least squares means for each of the three groups, and differences between them, and back-transformed the resulting estimates in order to report estimated differences on the original data scale. We repeated the analyses of covariance after including other patient characteristics, such as education level, employment status, and blood pressure. As the results from these analyses with expanded adjustments were very similar to our primary adjusted analyses, we do not report them here. All analyses were performed with SAS statistical software, version 9.4 (SAS, Inc., Cary, NC).

## Results

### Study population

The characteristics for all study participants are shown in Table 1. Patients in the APD group were the youngest. Compared with the CKD and the HD groups, the APD group had higher proportions of women and African-Americans, with high school education. Notably, most of the HD patients were unemployed. Overall comparisons among the groups resulted in significant differences also for glucose levels (*p* < 0.001), SBP (*p* = 0.03) and DBP (*p* = 0.03), and the number of antihypertensive medications (*p* = 0.002). For these variables, HD had significantly higher glucose levels than APD and CKD. APD patients had significantly less SBP than CKD patients and significantly higher DBP than HD patients, while APD patients took significantly fewer antihypertensive medications than CKD and HD patients. Although comparisons did not reach statistical significance, the APD group participants tended to be less obese and with a lower waist circumference compared to CKD and HD patients. APD patients had significantly (*p* = 0.003) smaller neck circumference compared to HD patients.

**Table 1:** Characteristics of study population

	APD (*N* = 22)	CKD 3b-5 (*N* = 89)	HD (*N* = 75)	*p* value
Age (years)	37.5 (31.5, 58.3)	51 (42.5, 64.0)	57.5 (46.0, 67.2)	0.001 HD > CKD^c^ CKD > APD^b^ HD > APD^a^
Women	16 (72.7 %)	29 (32.6 %)	25 (33.8 %)	0.002 CKD > APD^b^ HD > APD^b^
Whites	11 (50 %)	70 (78.7 %)	45 (60.8 %)	0.006 CKD > HD^b^ CKD > APD^b^
High school education	21 (95.5 %)	82 (92.1 %)	63 (85.1 %)	<0.001
Employed	9 (40.9 %)	40 (44.9 %)	11 (14.9 %)	<0.001 CKD > HD^a^ APD > HD^b^
Smoking				0.12
Current	5 (22.7 %)	13 (14.6 %)	8 (10.8 %)	
Former	11 (50 %)	28 (31.5 %)	33 (44.6 %)	
Never	6 (27.3 %)	48 (53.9 %)	33 (44.6 %)	
BMI (kg/m^2^)	23.8 (22.6, 28.9)	27.6 (25.0, 31.2)	27.2 (23.5, 31.2)	0.14
Waist circumference (cm)	93 (86.1, 112.9)	102 (90.75, 111.5)	105 (94.0, 115.8)	0.3
Neck circumference (cm)	36.5 (33, 39.4)	38 (36.0, 41.5)	40 (37.2, 43.7)	0.003 HD > APD^b^
Systolic blood pressure (mmHg)	132 (123, 148)	148.3 (132.0, 165.5)	145.7 (126.6, 169.4)	0.03 CKD > APD^b^
Diastolic blood pressure (mmHg)	90.8 (72.3, 96.5)	83.8 (73.4, 90.1)	78.5 (70.0, 88.9)	0.03 CKD > HD^c^ APD > HD^b^
Glucose (mg/dL)	95.3 (86.5, 114.4)	93.7 (985.0, 121)	119 (96.5, 147.2)	<0.001 HD > CKD^a^ HD > APD^b^
Total number of antihypertensives	1 (1, 2.5)	3 (2, 4)	2 (1, 3)	0.002 CKD > HD^b^ CKD > APD^c^
Diabetic nephropathy	3 (13.6 %)	29 (32.6 %)	24 (32 %)	
Hypertension	5 (22.7 %)	15 (16.9 %)	14 (18.7 %)	
Glomerulonephritis	8 (36.4 %)	16 (18 %)	11 (14.7 %)	
Other or unknown causes	6 (27.3 %)	29 (32.5 %)	26 (34.6 %)	

Results are presented as medians and interquartile ranges or as percentages. Numbers in parentheses reflect the 25th and the 75th percentile of the variables. Kruskal-Wallis test was used for the comparison of the distributions along all three study groups. Mann-Whitney *U* test was performed for all pairwise comparisons

^a^ *p* < 0.001; ^b^ *p* < 0.01; ^c^ *p* < 0.05 for the pairwise comparisons

The APD patients had been on cycler-assisted PD for a median of 10 (6, 16) months, while the HD patients had been on thrice weekly in-center HD for a median of 21.5 (9, 49.8) months. All dialysis patients received adequate dialysis dose. For the HD patients, the median single-pool Kt/V was 1.6 (1.5, 1.8) and the median urea reduction ratio (URR) was 73 (69.5, 76). For the APD patients, the median total weekly Kt/V was 2.34 (1.72, 3.15). The CKD sample had a median eGFR of 17.9 (13.1, 23.3) ml/min/1.73 m^2^, and a median serum creatinine of 4.0 (3.1, 4.9) mg/dl. The cause of CKD/ESRD is also presented in Table 1. The most common cause of renal dysfunction was diabetic nephropathy (CKD and HD) and glomerulonephritis (APD).

### Subjective and objective sleep characteristics

Continuous parameters of sleep, SDB, PLMS, SQ, depression, and their unadjusted differences across study groups are shown in Table 2. Compared with the HD group, the CKD patients had significantly greater TST and SE. The APD group had significantly greater stages 3 to 4 sleep compared to the CKD patients. Notably, median AHI was significantly higher in the HD group compared with the other two groups, but did not differ between the CKD and the APD patients. APD patients revealed the least percentage of TST with nocturnal hypoxemia. Finally, both HD and APD patients reported significantly poor SQ (high total PSQI score) compared to the CKD patients. There were no significant differences in stage 1, stage 2, REM sleep, micro-arousal index, and PLMI across all three study groups.

**Table 2:** Objective, subjective sleep parameters, and depression among study groups

	APD (*N* = 22)	CKD 3b–5 (*N* = 89)	HD (*N* = 75)	*p* value
Total sleep time (minutes)	342.7 (271.7, 475.3)	366.3 (298.3, 433.2)	313.3 (216.2, 388.2)	0.002 CKD > HD^a^
Sleep efficiency (%)	78.3 (57.6, 87.1)	77.8 (67.3, 85.1)	69.8 (59.5, 78.9)	0.018 CKD > HD^b^
Stage 1 (% of TST)	7.2 (4.7, 12.4)	10.1 (6.2, 15.9)	11.6 (6.8, 18.1)	0.11
Stage 2 (% of TST)	56.3 (48, 65.9)	61.2 (53.9, 69.8)	57.5 (51.0, 67.0)	0.13
Stages 3 and 4 (% of TST)	14.0 (4.3, 23.4)	5.4 (1.3, 10.9)	7.3 (1.3, 18.5)	0.009 APD > CKD^b^
Rapid eye movement sleep (% of TST)	20.9 (15.8, 25.6)	20.3 (14.5, 26)	17.9 (11.2, 22.3)	0.11
Apnea-hypopnea index	6.0 (2.3, 22.2)	8.8 (3.2, 27.6)	18.2 (6.7, 30.2)	0.008 HD > CKD^c^ HD > APD^c^
Obstructive apnea index	4.9 (1.8, 18.2)	6.2 (2.0, 17.3)	11.3 (4.1, 23)	0.034 HD > CKD^b^ HD > APD^b^
Nocturnal hypoxemia (≥3 % of TST)	1.1 (0.5, 6,7)	3.8 (1,5, 10)	1.5 (0.9, 8,5)	0.01 CKD > APD^b^ CKD > HD^b^
Micro-arousal index	9.2 (4.7, 13.4)	8.8 (3.9, 15.5)	8.5 (5.4, 14.0)	0.98
Periodic limb movement index	5.7 (1.6, 10.7)	2.4 (0.9, 5.3)	2.7 (0.7, 6.2)	0.14 APD > CKD^c^
Pittsburgh Sleep Quality Index score	8 (6.5, 13)	6 (4, 9)	8 (5, 11)	0.007 APD > CKD^b^ HD > CKD^c^
Patient Health Questionnaire-9 score	11 (8, 14.75)	10 (7, 13)	8 (6.25, 12)	0.08

Results are presented as medians and interquartile ranges or as percentages. Numbers in parentheses reflect the 25th and the 75th percentile of the variables. Stages 1, 2, 3, and 4, and rapid eye movement sleep are expressed as percent of total sleep time. Apnea hypopnea Index, obstructive apnea index, micro-arousal index, and periodic limb movement index are expressed as events per hour of total sleep time

*Sleep efficiency* percentage of total sleep time as a proportion of the total study duration, *Apnea hypopnea index*, number of apneas/hypopneas during total sleep time, *Obstructive apnea index* number of obstructive apneas per hour of total sleep time, *Nocturnal hypoxemia* ≥3 % total sleep time, percentage of subjects who had at least 3 % of total sleep time with oxyhemoglobin saturation less than 90 %, *Micro-arousal index* number of microarousals/hour of sleep, *Periodic limb movement index* number of periodic limb movements per hour of total sleep time

^a^ *p* < 0.001; ^b^ *p* < 0.01; ^c^ *p* < 0.05 for the pairwise comparisons

### Depression results

Results from the PHQ-9 questionnaire and their unadjusted differences across study groups are also shown in Table 2. APD patients reported the highest total PHQ-9 score (moderately severe depression). However, the among-group comparison was not statistically significant.

### Multivariable results

**Fig. 1:**
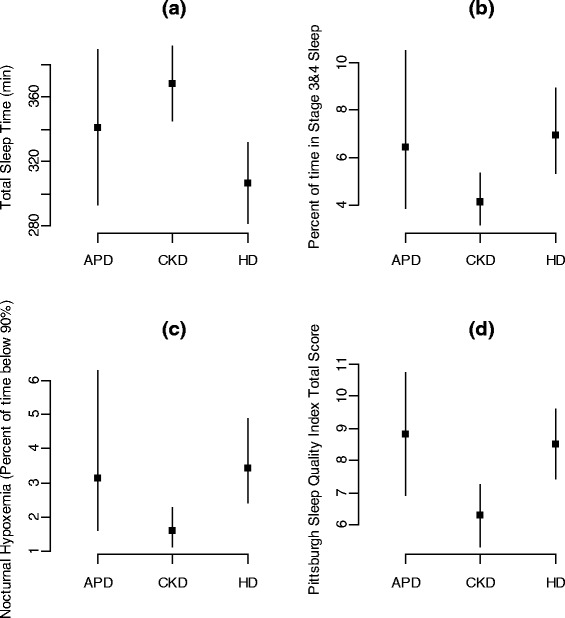
Multivariate analyses results. Least squares means and 95 % confidence intervals for sleep parameters with significant differences among groups after adjustment for age, gender, race, and BMI. **a** Estimates of total sleep time. **b** Estimates of the percentage of TST in stages 3 and 4 of sleep. **c** Estimates of the percentage of TST with oxygen saturation less than 90 % (nocturnal hypoxemia). **d** Estimates of sleep quality (total score of PSQI)

After adjusting for age, sex, race, and BMI, significant differences among groups were apparent for TST (*p* = 0.003), stages 3 to 4 sleep (*p* = 0.018), percentage of TST with nocturnal hypoxemia (*p* = 0.009), and PSQI scores (*p* = 0.005) as shown in Table 3. Further adjustment for additional covariates resulted in minimal changes from the simpler adjustments and are therefore not shown. Differences among groups after adjustment are illustrated in Fig. 1, which shows estimates of least squares means and 95 % confidence intervals for the estimates. CKD patients had significantly more TST than HD patients. They also spent a significantly lower proportion of TST in stages 3 to 4 sleep and experienced less percent of TST with nocturnal hypoxemia than HD patients. Both APD and HD patients had higher average PSQI scores than CKD patients. No other comparisons of sleep parameters among groups reached statistical significance.

**Table 3:** Differences in objective, subjective sleep parameters, and depression between each dialysis group and the CKD group, after adjustment for age, gender, race, and BMI; 95 % confidence intervals are also shown

	APD-CKD 3b–5	HD-CKD 3b–5	*p* value
Total sleep time (minutes)	−26.8 (−80.9 to 27.2)	−61.6 (−96.3 to −27.0)	0.003
Sleep efficiency (%)	−4.05 (−11.67 to 3.57)	−4.36 (−9.26 to 0.54)	0.18
Stage 1 (% of TST)	0.66 (−2.99 to 4.31)	1.17 (−1.23 to 3.58)	0.63
Stage 2 (% of TST)	−4.82 (−11.60 to 1.96)	−5.02 (−9.36 to −0.68)	0.057
Stages 3 and 4 (% of TST)	2.34 (−0.69 to 5.38)	2.81 (0.80 to 4.81)	0.018
Rapid eye movement sleep (% of TST)	0.41 (−4.83 to 5.65)	−1.36 (−4.63 to 1.92)	0.66
Apnea hypopnea index	0.92 (−4.92 to 6.77)	4.84 (0.47 to 9.21)	0.091
Obstructive apnea index	1.25 (−3.51 to 6.02)	2.88 (−0.40 to 6.15)	0.22
Nocturnal hypoxemia (≥3 % of TST)	1.54 (−0.24 to 3.32)	1.82 (0.63 to 3.00)	0.009
Micro-arousal index	1.19 (−2.90 to 5.29)	0.29 (−1.96 to 2.54)	0.84
Periodic limb movement index	2.04 (0.25 to 3.82)	0.46 (−0.50 to 1.41)	0.078
Pittsburgh Sleep Quality Index score	2.54 (0.37 to 4.71)	2.22 (0.74 to 3.71)	0.005
PHQ-9 score	1.51 (−0.48 to 3.50)	−0.13 (−1.30 to 1.03)	0.26

Results are presented as differences in least squares means between the identified groups. Numbers in parentheses reflect the endpoints of the 95 % confidence interval for the indicated group difference

## Discussion

To our knowledge, this is the first study to examine objectively and subjectively measured sleep disturbances and self-reported presence of symptoms of depression across APD patients and to compare them to CKD and HD patients. In our study, sleep efficiency and sleep architecture were relatively preserved despite being on APD treatment. Sleep parameters of SDB were not significantly different among study groups after accounting for differences in age, gender, race, and BMI. Furthermore, PLMS were not significantly elevated among the study groups, while self-reported SQ was poor among dialysis patients, especially those on HD. Finally, depression was equally present among all study groups.

PSG is the gold standard for diagnosing SDB and PLMS in APD patients. According to our multivariate analyses findings, median AHI, moderate to severe SDB, and nocturnal hypoxemia were higher but not significantly different in the APD and the HD groups compared with the advanced CKD group. These objective measures of SDB are consistent with previous work comparing sleep parameters between chronic HD and CAPD patients by subjective means. According to this study, no differences were seen between HD and PD patients in characteristics of sleep problems [37]. It should be noted that the rate of moderate to severe sleep apnea in our study cohort is lower, and this is likely due to the APD patients being younger and thinner and having a higher proportion of women than in previous work.

Volume overload comprises a major problem for the PD patients, especially those with minimal or no residual renal function. In addition, dry weight in PD is difficult to achieve. An edema-free state is typically utilized as the target of volume control. However, PD patients can be free of edema despite significant volume overload [38]. No objective measurements (chest ultrasound, bioelectrical impedance analysis, or neck and chest MRI) of volume status were included in the initial study design. The only available information from the APD patients that could be related to a gross but not absolutely reliable estimate of their volume status are neck and waist circumference, the fact that most had residual renal function and were free of external anasarca or pedal edema.

In our multivariate analyses, APD patients had more PLMS but not significantly different compared to all other patients. However, it is thought that PLMS may affect up to 50–70 % of patients with ESRD [12] and this data measuring PLMI provides essential information for physicians demonstrating that PLMS are also found among patients undergoing APD. In a study by Jung et al. [14], PLMI was associated with both a poor cardiovascular outcome and mortality in maintenance HD patients, while in a study by Lindner et al. [9], PLMS were associated with stroke and cardiovascular risk factors in patients with ESRD.

In both univariate and multivariate analyses, we found that patients on HD and on APD had poorer sleep than patients with CKD. Although patients with HD had slightly poorer sleep than patients with APD, we were unable to conclude that there were significant differences between these two groups after multivariate adjustment. Being on HD has been associated with poor SQ [27], and in our study, HD patients had slightly worse self-reported SQ compared to the APD patients. The clinical importance and urgency of this difference in subjective SQ is evidenced by a high prevalence of sleep-promoting medication use among dialysis patients [39]. In addition, poor SQ has been associated with an increased prevalence of cardiovascular disease. Although the causes are multifactorial and incompletely understood, the detection and treatment of SQ in dialysis patients may have a significant impact on clinical outcomes, since self-reported sleep problems have strongly been linked to disability days, health care utilization, and QOL [40] as well as the ability to function [41]. Finally, addressing SQ in APD patients may be particularly important since poor SQ may cause some patients to switch dialysis modality.

Depression is the most common psychological problem presented by dialysis patients, and in our study, moderate depression was equally present in all study groups. Our findings are in accordance to a study by Losso et al. [42] where depression was compared between HD, APD, and CAPD patients. They found no differences in depression among the modalities. The psychological evaluation of the patient receiving a home-performed dialysis therapy such as APD is extremely important for two reasons. First, there is a significant association between depression scores on standardized questionnaires and patients outcomes, such as hospitalizations and mortality [43, 44]. Second, a prompt and effective therapy will maximize a patient’s health-related quality of life (HRQOL) and well-being [45]. Disturbed sleep, depressed mood, and diminished HRQOL can also be a potential consequence of persistent pain among dialysis patients [46, 47].

The results of our study should be interpreted after taking into account certain limitations. First, the APD group was demographically and clinically distinct from the other study groups. Therefore, it is difficult to uniquely ascribe the observed differences in sleep parameters among the study groups to group membership itself because of the presence of differences which may confound the results. However, the literature describing sleep disorders tends to underrepresent the APD group. Second, this was a single-center study and this may limit the generalizability of our conclusions as well. However, the patients were receiving standard of care and this report provides important information to providers and describes sleep among women on APD. Third, the sample size was relatively small and provided limited statistical power to detect significant differences between groups. With 22 APD patients, we had 80 % power to detect differences of just over 2/3 of a standard deviation in magnitude between APD patients and each of the two other study groups. These moderate-to-large detectable differences limited our ability to rule out the presence of smaller differences among groups. The limited power to protect against false negative findings must also be weighed against the possibility of false positive findings, as we have performed a large number of statistical tests and this can lead to spurious significant findings due to multiple testing. Finally, no objective measures of volume overload were included in the initial study design. Despite these limitations, this study contributes to the development of meaningful understanding of the relationship between treatment of kidney failure, SDB, SQ, and depression, particularly given the limited amount of sleep data available from APD patients.

Given the complexity of causes of fatigue and poor sleep in this population, PSG is needed to determine the full range of potential sleep disorders that may disrupt sleep and impair daytime function. Given also the nature of dialysis treatment, it is not possible to use a cross-over design where patients serve as their own controls. This obstacle was addressed by comparing the APD patients with two different groups of patients, one under a different dialysis modality and one with advanced kidney dysfunction not yet on dialysis. Despite the population disparity, the results presented herein could partly explain the significance of age and BMI to characterize the presence of SDB and is in accordance to previous studies [48–51]. Finally, most studies in the past examining sleep in larger PD cohorts used subjective measures (questionnaires, sleep diaries) and PSG has been performed only in small cohorts of APD patients. This is the main strength of our work.

## Conclusions

In conclusion, this study indicates that APD patients may have similar sleep parameters and sleep architecture compared to the other groups despite undergoing exchanges overnight. Older age and higher BMI were the main determinants of moderate to severe SDB and nocturnal hypoxemia among study groups, providing important risk factors for physicians to consider when referring patients for sleep evaluation. Since the PD population is growing fast, future work should examine the impact of treating SDB, poor SQ and depression on functioning, cardiovascular health, and mortality in this high risk population.

## Abbreviations

AHI: apnea-hypopnea index
APD: automated peritoneal dialysis
CKD: chronic kidney disease
ECG: electrocardiogram
EEG: electroencephalogram
eGFR: estimated glomerular filtration rate
EMG: electromyogram
EOG: electrooculogram
ESRD: end-stage renal disease
HD: hemodialysis
NREM: non-rapid eye movement
PD: peritoneal dialysis
PHQ-9: Patient Health Questionnaire-9
PLMI: Periodic Limb Movement Index
PLMS: periodic limb movements
PSG: polysomnography
PSQI: Pittsburgh Sleep Quality Index
REM: rapid eye movement
SDB: sleep-disordered breathing
SQ: sleep quality
URR: urea reduction ratio
